# Relationship status and gender-related differences in response to infidelity

**DOI:** 10.3389/fpsyg.2023.1158751

**Published:** 2023-05-24

**Authors:** Tsukasa Kato, Nobutoshi Okubo

**Affiliations:** ^1^Laboratory of Stress Sciences, Department of Psychology, University of Human Environments, Matsuyama, Ehime, Japan; ^2^Institute of Human Sciences, Toyo University, Tokyo, Japan

**Keywords:** infidelity, jealousy, sexual imagination hypothesis, gender similarity, 2D:4D digit ratio, evolutionary psychology

## Abstract

**Introduction:**

The sexual imagination hypothesis suggests that responses to a partner’s infidelity emerge from the sociocultural factors that affect individuals’ imagining of that occurrence irrespective of biological sex, including relationship status (i.e., the experience of a serious, committed relationship). Nevertheless, evolutionary psychological perspectives predict that responses to a partner’s infidelity emerge from a sex-specific evolved innate mechanism.

**Methods:**

A lower 2D:4D digit ratio is associated with more robust responses to a partner’s sexual infidelity. In this study, participants (660 males and 912 females) were requested to measure finger lengths, reactions to their partners’ sexual and emotional infidelity, and relationship status.

**Results:**

A logistic regression and multiple regression analyses revealed that relationship status was uniquely associated with responses to a partner’s sexual and emotional infidelity beyond the effects of sex and 2D:4D. Those in committed relationships were more upset or distressed over their partners’ infidelity, particularly over sexual infidelity, than those not in committed relationships.

**Discussion:**

The results supported the sexual imagination hypothesis indirectly, while evolutionary psychological perspectives were met with skepticism. Our findings implied that sex differences in jealousy result from relationship status, and that responses to partners’ infidelity are more alike than different.

## Introduction

The research on sex differences in responses to a partner’s infidelity (hereafter, jealousy), led and developed by evolutionary psychological perspectives, hypothesizes that sex differences in jealousy emerge owing to innate sex-specific mechanisms. Additionally, the empirical studies on sex differences in jealousy have provided crucial evidence supporting evolutionary psychology (Buss, 2018). However, sociocultural perspectives on sex differences (or similarities) in jealousy predict that sex differences (or similarities) arise through the acquisition of culturally sex-specific constructs, and have provided evidence for skepticism regarding evolutionary psychology perspectives. This conflict began to intensify around the 2000s and remains unresolved (Kato, 2022b). To facilitate understanding of our hypothesis, which is based on sociocultural perspectives and their findings, we will first describe the evolutionary psychological perspective.

### Sex differences in responses to a partner’s infidelity and 2D:4D digit ratio

According to the evolutionary psychological perspective, males are more sensitive to (upset or distressed by) their partners’ sexual infidelity than females. In contrast, females are more sensitive to their partners’ emotional infidelity than males (Buss, 2018). The sex differences in jealousy are rooted in human ancestry. Specifically, ancestral males were wary of their partners’ potential sexual contact with other males. In contrast, ancestral females were free from the risk of maternal uncertainty. However, a partner’s emotional infidelity poses a threat as it could lead to a loss of paternal investment and resources. These resources may be diverted to a rival female and her children (Buss, 2018). This evolutionary psychological perspective is the sex-specific evolved jealousy mechanism (EJM).

To test the EJM hypothesis, Buss and his colleagues (Buss et al., 1992) required college students to choose whether they would be more upset or distressed by their partner’s sexual infidelity (i.e., enjoying passionate sexual intercourse with another person) or emotional infidelity (i.e., forming a deep emotional attachment to another person). The results revealed that 60% of male students opted for the sexual infidelity scenario, while 83% of female students chose the emotional infidelity scenario (Study 1). These findings supported the EJM hypothesis. Moreover, the EJM hypothesis has been repeatedly supported in studies performed worldwide employing Buss et al.’s (1992) paradigm (Buss, 2018). For instance, some evolutionary psychologists (Edlund and Sagarin, 2017) asserted that specific meta-analyses provided strong evidence for the sex differences in jealousy.

Nevertheless, these findings are only indirect evidence for the EJM hypothesis. According to the EJM hypothesis, sex differences in jealousy have biological origins. Therefore, the relationship between sex differences in jealousy and biological mechanisms must be examined to test the EJM hypothesis. Among these biological mechanisms, the 2D:4D digit ratio may be one. Although some studies have tested the EJM hypothesis using physiological responses as a marker of jealousy, such as heart rate, startle eyeblink, and brain activities, using functional magnetic resonance imaging (see Kato, 2022b), to our knowledge, no study has examined the relationship between sex differences in jealousy and biological mechanisms except the 2D:4D research. The second to fourth-digit ratio has been used to indicate prenatal androgen. This ratio is higher in females than males (Hönekopp and Watson, 2010). Furthermore, 2D:4D has been reported to be associated with behavioral traits, such as personality, cognitive abilities, sexual orientation, sports performance, and risk of illnesses (Leslie, 2019). Most 2D:4D studies were published in psychology departments (Voracek and Loibl, 2009). Furthermore, sex differences related to 2D:4D have been used as evidence to support specific evolutionary psychological perspectives (e.g., Gallup and Frederick, 2010, for a review).

Examining the relationship between 2D:4D and sex differences in jealousy may contribute to comprehending biological mechanisms’ influence on it. Maner et al. (2014) proposed that, based on a psychological perspective called *fast life history strategies*, the masculinizing effects of prenatal testosterone bring early investment in reproduction and behaviors to compete directly with intrasexual rivals to ensure immediate reproductive access to potential mates. Consequently, greater exposure to prenatal testosterone (lower 2D:4D) might potentiate a heightened propensity to respond competitively and aggressively toward possible rivals, particularly when encountering the threat of infidelity. In addition, Maner et al. (2014) demonstrated that lower 2D:4D was associated with greater muscle flexion (representing oppositional and confrontational behaviors) when imagining one’s partner’s infidelity with an attractive rival (i.e., flirting with being intimate with another person at a party). Based on this, Fussell et al. (2011) hypothesized that individuals in lower 2D:4D might be more upset or distressed by their partner’s sexual infidelity than emotional infidelity in both sexes; they tested this hypothesis in heterosexual undergraduates and postgraduates. Another study (Bendixen et al., 2015) on heterosexual undergraduates replicated their findings. However, only these two studies examined the relationship between 2D:4D and jealousy.

In conclusion, regarding the relationship between 2D:4D and jealousy, an evolutionary psychological perspective predicts that individuals in lower 2D:4D will be more upset or distressed by their partner’s sexual infidelity in both sexes.

### Relationship status and sex differences in jealousy

Some sociocultural perspectives exhibit skepticism regarding the EJM hypothesis (see Kato, 2022b), such as relationship status (i.e., the experience of a serious, committed relationship). According to the EJM hypothesis, sex differences in jealousy should be observed regardless of status. Sex differences by relationship status should be more significant for those who have experienced a serious, committed relationship than those who have not.

However, some studies (e.g., Becker et al., 2004; Guadagno and Sagarin, 2010; Kato, 2014a, 2021; Pazhoohi et al., 2019) found that sex differences in jealousy were due to the relationship status, but not innate mechanism (i.e., EJM); therefore, sociocultural perspectives regard this phenomenon as sex differences (similarities), instead of sex differences in jealousy. Kato (2014b) found no sex differences in jealousy among male and female college students who were or had been in a serious, committed relationship using a large sample (*n* = 2,241). Sex differences in jealousy were observed exclusively in college students who were not in serious, committed relationships (i.e., men were more upset over sexual infidelity, and women were more upset over emotional infidelity). Specifically, female college students in a serious, committed relationship were more upset or distressed over sexual infidelity than those who were not in a serious, committed relationship; in contrast, male college students in a serious, committed relationship were more upset or distressed over emotional infidelity than those who were not in a serious, committed relationship. For the former sample (i.e., female college students in a serious, committed relationship), the Type II error probability of falsely accepting an incorrect null hypothesis was low (1–β = 0.956, effect size partial *η*^2^ = 0.005). This finding indicated that the probability that the null hypothesis (no sex differences) was accepted falsely was low. It implied that the result of no sex differences in jealousy is highly reproducible.

Kato (2014b) explained these findings that participants in a serious, committed relationship could imagine their partners’ infidelity (especially sexual infidelity for female college students) more readily and vividly than participants who were not in a serious, committed relationship. As explained by Kato (2014b), some studies (e.g., Becker et al., 2004; Kato, 2014a, 2021) found that individuals in a committed relationship more easily imagine their partners’ infidelity than those who are not. This phenomenon is also observed when other sexual stimuli than sexual infidelity are used. Specifically, individuals in a committed relationship strongly respond to sexual stimuli regardless of sex than those who are not (see Kato, 2021). This phenomenon can explain by the sexual imagination hypothesis (Harris, 2000; Kato, 2014a, 2017, 2022b). According to the sexual imagination hypothesis, apparent sex differences in jealousy emerge owing to the differences in vivid imagination between men and women, but not the EJM. Therefore, the sex differences in jealousy are not observed when both men and women explicitly imagine their partners’ infidelity, especially sexual infidelity. Generally, men can envision sexual infidelity more explicitly or easily than women, while women can envision emotional infidelity more explicitly or easily than men. More specifically, the former difference is recognized as significant (Kato, 2014a, 2022b). This phenomenon is also observed when other sexual stimuli than sexual infidelity are used (Kato, 2022a). Some studies (Harris, 2000; Kato, 2014a,b, 2021, 2022a) demonstrated this sexual imagination hypothesis. Based on Kato’s (2014b) explanation described above, for example, the experience of being cheated on by a partner enhanced the imaging of sexual infidelity for those involved in serious, committed relationships. Frederick and Fales (2016) showed that individuals who experienced their partners’ unfaithfulness were upset over sexual infidelity compared to those who had previously not experienced this life event.

Most studies (e.g., Becker et al., 2004; Guadagno and Sagarin, 2010; Kato, 2014b, 2021) demonstrated that relationship status could explain sex differences in jealousy using a continuous measurement paradigm. In contrast, only a few studies (e.g., Kato, 2014a) used a forced-choice measurement paradigm. The *forced-choice measurement paradigm* is the method proposed by Buss et al. (1992), in which participants choose the more upsetting or distressing of the infidelity types (sexual or emotional infidelity). The *continuous measurement paradigm* is a method in which participants rate the degree to which they were upset or distressed by each infidelity type. In studies without specific participants, those using a forced-choice measure were more likely to support the EJM hypothesis. Studies using a continuous measure were more likely to reject the EJM hypothesis. A meta-analysis (*k* = 168, *N* = 125,698; Kato, 2017) incorporating the largest sample among those showed that approximately 69.2% of the studies using forced-choice measurement supported the EJM hypothesis. In contrast, approximately 66.5% of the studies using continuous measurement provided evidence that the EJM hypothesis should be viewed skeptically (Kato, 2022b). Therefore, the present study tested the sexual imagination hypothesis and the EJM hypothesis using forced-choice and continuous measures.

Based on the sexual imagination hypothesis, we hypothesized that relationship status would be associated with jealousy beyond the effects of biological sex and 2D:4D on jealousy. Specifically, individuals in committed relationships would be more upset or distressed by their partner’s infidelity than those not in a committed relationship, regardless of biological sex and 2D:4D. Such a trend would be strongly observed in a partner’s sexual infidelity. Our study differs from many previous studies related to the sexual imagination hypothesis in that we attempted to demonstrate that the predictions based on it are valid for both forced-choice and continuous measurement paradigms. Our study also differs from previous studies related to the sexual imagination hypothesis in that we measured 2D:4D This measurement demonstrated a biological mechanism of sex differences in jealousy.

## Methods

### Participants and procedure

Participants were recruited through lectures at colleges in Japan. Participants comprised 660 males and 912 females (biological sex) aged 30 and younger (18 and 29 years, mean age = 19.88, SD = 1.40), who were heterosexuals and were recruited from colleges in Japan. We recruited heterosexuals exclusively because interpretations of sex differences in jealousy in homosexual individuals, in evolutionary psychological and sociocultural perspectives, differ from those in heterosexuals (see Kato, 2022b). Eleven students did not respond to questions about their biological sex or sexual orientation. The age of 30 years or younger is consistent with Bendixen et al.’s (2015) criteria, which was used in examining the relationships between sex differences in jealousy and 2D:4D to replicate the previous studies (Fussell et al., 2011; Bendixen et al., 2015). Additionally, participants reported being (or had been) in a serious, committed relationship; based on Kato’s (2014a) classification, casual dating was excluded from a serious, committed relationship. According to the evolutionary psychological perspective, long-term mating strategies (used in serious, committed relationships) differ from short-term ones (used in casual dating).

After provided written informed consent, participants answered sociodemographic questions, including sex and age. They answered the questionnaire, and then their finger lengths were measured.

### Measures

All instructions, questionnaires, and measures were provided in Japanese.

#### Responses to partner’s infidelity (jealousy)

Jealousy was measured using forced-choice and continuous measures. The order in which these two paradigms were presented was random. In the forced-choice measurement paradigm, participants were required to select one of the following scenarios in which they would be more upset or distressed: (a) your partner forming a deep emotional attachment to that person (i.e., emotional infidelity) and (b) your partner enjoying passionate sexual intercourse with the other person (i.e., sexual infidelity). This method was identical to one proposed by Buss et al. (1992). The score calculated by the forced-choice measurement is referred to as the F-C jealousy score in this study.

In the continuous measurement paradigm, participants were required to rate the degree to which their partners’ sexual and emotional infidelity would upset or distressed them, using six-point Likert-type scales ranging from 1 (not at all upset or distressed) to 6 (extremely upset or distressed). In this study, the scores calculated by the continuous measurement for sexual and emotional infidelity are referred to as the sexual and emotional jealousy scores, respectively.

#### 2D:4D digit ratio

Based on Ribeiro et al. (2016) recommendations, participants’ finger lengths were measured directly. Ribeiro et al. (2016) mentioned that direct 2D:4D tends to be greater than indirect. Furthermore, it is more strongly associated with target traits than indirect 2D:4D. In this study, a digital caliper (TDN-100, TRUSCO, Pro Tool, Japan), calibrated to the nearest 0.01 mm with instrumental error ± 0.003 mm, was used to measure finger length. Out of 1,572 participants, 1,426 were right-handed (90.7%). The final 2D:4D ratios were calculated by dividing 2D by 4D length.

#### Relationship status

Out of 1,572 participants, 983 reported being (or had been) in a serious, committed relationship at the time of this study (62.5%). The remaining 589 participants reported not being in a serious, committed relationship. Casual dating was excluded from a serious, committed relationship. Participants in committed relationships had a mean (SD) and median duration relationship of 12.10 (12.54) and 8 months, respectively.

### Data analysis

To test our hypothesis, a logistic regression analysis on an F-C jealousy score was conducted with sex, 2D:4D, and relationship status scores (Step 1) and an interaction score between sex and relationship status scores (Step 2) as predictors of an F-C jealousy score. Second, hierarchical multiple regressions on each score of sexual and emotional jealousy were conducted with sex, 2D:4D, and relationship status scores (Step 1) and an interaction score between sex and relationship status scores (Step 2) as predictors of each score of sexual and emotional infidelity.

## Results

Table 1 shows the frequencies of an F-C jealousy score, the means and standard deviations of a 2D:4D score, and sexual and emotional infidelity scores by sex and relationship status.

**Table 1:** Means and standard deviations of imaginations and responses to partners’ infidelity by sex and relationship status.

Variable	Frequency		Frequency		*χ*^2^ value	*p* value	Effect size (φ)
	Emotional	Sexual	Emotional	Sexual			
Sex	Men (*n* = 660)		Women (*n* = 912)				
F-C jealousy	347	286	571	341	5.64	0.018	0.06
Relationship status	Presence (*n* = 983)		Absence (*n* = 589)				
F-C jealousy	519	464	426	163	58.58	<0.001	0.19
Variable	Mean	SD	Mean	SD	*t* value	*p* value	Effect size (*d*)
Sex	Men (*n* = 660)		Women (*n* = 912)				
Sexual jealousy	4.39	1.38	4.24	1.35	2.16	0.031	0.11
Emotional jealousy	4.54	1.25	4.53	1.25	0.14	0.889	0.01
Right 2D:4D	0.97	0.05	0.98	0.04	3.49	<0.001	0.17
Relationship status	Presence (*n* = 983)		Absence (*n* = 589)				
Sexual jealousy	4.68	1.24	3.67	1.32	15.19	<0.001	0.80
Emotional jealousy	4.83	1.09	4.04	1.35	12.68	<0.001	0.66
Right 2D:4D	0.98	0.04	0.97	0.05	−2.27	0.024	0.23

F-C jealousy is response in a partner’s infidelity that scored using the forced-choice measure (score range = 1 or 2). Sexual and emotional jealousy are responses in a partner’s sexual and emotional infidelity that scored using the continuous measure (score range = from 1 to 6), respectively. Presence and absence of relationship status are participants in a committed relationship and those not in one, respectively.

A logistic regression analysis on an F-C jealousy score, conducted to test our hypothesis, revealed that the model at Step 2 was significant (Table 2): χ^2^(4) = 74.66, *p* < 0.001, Nagelkerke *R*^2^ = 0.063. In addition, the significant interaction (*B* = 0.61, *SE* = 0.23, Wald = 7.25, *p* = 0.007, odds ratio [OR] = 1.84, 95% confidence interval [CI] for OR = 1.18, 2.88) and the effect of exclusively relationship status (*B* = 1.14, *SE* = 0.16, Wald = 54.21, *p* < 0.001, OR = 3.13, 95% CI for OR = 2.31, 4.24) were found. The effect of relationship status indicated that participants in a serious, committed relationship were more upset or distressed over their partners’ sexual infidelity than those not in one. Follow-up analysis of the interaction between sex and relationship status revealed that both male (47.9% vs. 35.4%; χ^2^(1) = 9.63, *p* = 0.002, φ = 0.12) and female (46.7% vs. 22.3%; χ^2^(1) = 54.63, *p* < 0.001, φ = 0.25) participants in committed relationships chose sexual infidelity as more upsetting or distressing than those not in one. Additionally, the effect of sex was significant only in participants not in committed relationships; males chose sexual infidelity as more upsetting or distressing than females (35.4% in males vs. 22.3% in females), and more females chose emotional infidelity than males (64.6% in males vs. 77.7% in females): χ^2^(1) = 12.13, *p* < 0.001, φ = 0.14. However, no significant effect of sex was found among participants in committed relationships (47.9% in males vs. 46.7% in females who chose sexual infidelity): χ^2^(1) = 0.13, *p* = 0.722, φ = 0.01.

**Table 2:** Logistic regression analysis predicting the response to a partner’s infidelity when using a forced-choice measure (*N* = 1,572).

Predictor	B	SE	Wald	*p* value	Exp(B)	95% CI	
						LL	UL
Step 1							
Sex	−0.23	0.11	4.75	0.029	0.79	0.64	0.98
Right 2D:4D	−1.79	1.19	2.25	0.134	0.17	0.02	1.73
Relationship status	0.87	0.11	58.60	<0.001	2.38	1.91	2.97
χ^2^(3) = 67.39, *p* < 0.001, Nagelkerke *R*^2^ = 0.057							
Step 2							
Sex	−0.03	0.13	0.06	0.804	0.97	0.75	1.25
Right 2D:4D	−1.90	1.20	2.53	0.112	0.15	0.01	1.56
Relationship status	1.14	0.16	54.21	<0.001	3.13	2.31	4.24
Interaction	0.61	0.23	7.25	0.007	1.84	1.18	2.88
χ^2^(4) = 74.66, *p* < 0.001, Nagelkerke *R*^2^ = 0.063							

CI is confidence interval; LL and UL are lower and upper limits, respectively. Response to a partner’s infidelity (emotional infidelity = 1 and sexual infidelity = 2). Sex (male = 1 and female = 2). Relationship status (participants in not a committed relationship = 1 and those in one = 2). Interaction is sex × relationship status.

**Figure 1:**
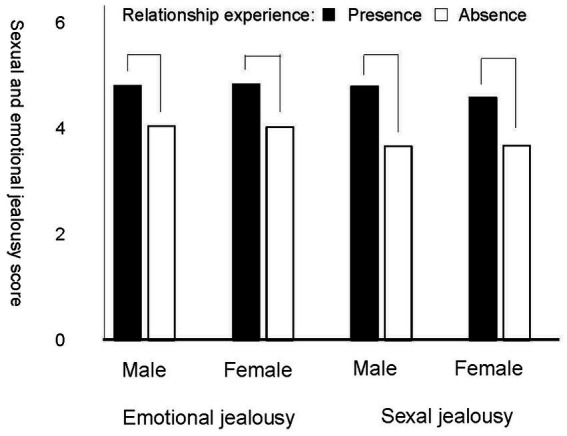
Sexual and emotional jealousy are responses in a partner’s sexual and emotional infidelity that scored using the continuous measure (score range = from 1 to 6), respectively. Presence and absence of relationship experience are participants in a committed relationship and those in not one, respectively. The results of statistical tests are based on an 2 (sex) × 2 (relationship experience) × 2 (type of jealousy; sexual vs. emotional jealousy) analysis of variance; all significant levels are *p* < 0.001.

A hierarchical multiple regression on a sexual jealousy score, conducted to test our hypothesis, revealed that the delta multiple correlation coefficient (Δ*R*) values at Step 2 were not significant (Table 3): Δ*R*^2^ = 0.01, Δ*F* (1,1567) = 2.63, *p* = 0.105, Cohen’s *f*^2^ = 0.01. However, the *R* value at Step 1 was significant: *R*^2^ = 0.13, *F* (3,1568) = 78.87, *p* < 0.001, Cohen’s *f*^2^ = 0.15; the significant effect of only relationship status was found: *β* = 0.36, *t* = 15.21, *p* < 0.001, indicating that both males and females in committed relationships were more upset or distressed by their partners’ sexual infidelity than those not in one. Figure 1 demonstrates the association of relationship status with sexual and emotional jealousy.

A hierarchical multiple regression on an emotional jealousy score revealed that the Δ*R*-value in Step 2 was not significant (Table 2): Δ*R*^2^ = 0.01, Δ*F* (1,1567) = 0.17, *p* = 0.678, Cohen’s *f*^2^ = 0.01. However, the *R*-value in Step 1 was significant: *R*^2^ = 0.09, *F* (3,1568) = 53.59, *p* < 0.001, Cohen’s *f*^2^ = 0.10; the significant effect of only relationship status was found: *β* = 0.31, *t* = 12.64, *p* < 0.001, indicating that males and females in committed relationships were more upset or distressed by their partner’s emotional infidelity than those not in one.

A *χ*^2^-test on an F-C jealousy score, conducted to test the EJM hypothesis, showed that males chose sexual infidelity as more upsetting or distressing than females (43.3% vs. 37.4%; χ^2^(1) = 5.64, *p* = 0.018, φ = 0.06). A *t*-test on the sexual jealousy score also showed that males reported being more upset or distressed by their partner’s sexual infidelity than females (*t*(1570) = 2.16, *p* = 0.031, *d* = 0.11). However, a *t*-test on the emotional jealousy score showed an insignificant sex difference.

A *t*-test conducted to determine the sex difference of right 2D:4D revealed that males’ 2D:4D was lower than that of females (*t*(1570) = 3.49, *p* < 0.001, *d* = 0.17). Furthermore, *t*-tests on an F-C jealousy score, conducted to assess the hypothesis of evolutionary psychological perspective regarding the association between 2D:4D and jealousy, showed a non-significant difference between 2D:4D in participants who chose sexual infidelity as more upsetting or distressing and 2D:4D in those who chose emotional infidelity among both males (*t*(658) = 1.15, *p* = 0.250) and females (*t*(910) = 0.19, *p* = 0.852). Additionally, the correlations of a 2D:4D score with sexual and emotional jealousy scores were insignificant among males (*r* = 0.05, *p* = 0.188 and *r* = −0.01, *p* = 0.756) and females (*r* = 0.04, *p* = 0.211 and *r* = 0.05, *p* = 0.160).

Additional analyses extracting only participants in a serious, committed relationship found no significant effects of relationship duration on sex differences in jealousy (Table 3).

**Table 3:** Hierarchical multiple regression analyses predicting responses to partner’s sexual and emotional infidelity when using a continuous measure (*N* = 1,572).

Predictor	B	95% CI		*t* value	*p* value
		LL	UL		
Sexual infidelity					
Step 1					
Sex	−0.12	−0.25	0.00	1.91	0.057
Right 2D:4D	−0.79	−2.14	0.55	1.15	0.249
Relationship status	1.01	0.88	1.14	15.21	<0.001
*R*^2^ = 0.13, *F* (3,1568) = 78.87, *p* < 0.001, Cohen’s *f*^2^ = 0.15					
Step 2					
Sex	0.01	−0.20	0.22	0.11	0.909
Right 2D:4D	−0.75	−2.09	0.60	1.09	0.277
Relationship status	1.35	0.92	1.79	6.07	<0.001
Interaction	−0.22	−0.48	0.05	1.62	0.105
Δ*R*^2^ = 0.01, Δ*F* (1,1567) = 2.63, *p* = 0.105, Cohen’s *f*^2^ = 0.01					
Emotional infidelity					
Step 1					
Sex	0.01	−0.11	0.13	0.13	0.898
2D:4D	−0.36	−1.69	0.97	0.53	0.598
Relationship status	0.79	0.67	0.91	12.64	<0.001
*R*^2^ = 0.09, *F* (3,1568) = 53.59, *p* < 0.001, Cohen’s *f*^2^ = 0.10					
Step 2					
Sex	−0.03	−0.22	0.17	0.25	0.801
2D:4D	−0.37	−1.70	0.96	0.54	0.588
Relationship status	0.71	0.30	1.12	3.38	<0.001
Interaction	0.05	−0.20	0.30	0.42	0.678
Δ*R*^2^ = 0.01, Δ*F* (1,1567) = 0.17, *p* = 0.678, Cohen’s *f*^2^ = 0.01					

CI is confidence interval; LL and UL are lower and upper limits, respectively. Sex (male = 1 and female = 2). Relationship status (participants not in a committed relationship = 1 and those in one = 2). Interaction is sex × relationship status.

## Discussion

We hypothesized that individuals in committed relationships would be more upset or distressed over their partners’ infidelity, especially sexual infidelity, than those not in committed relationships. Furthermore, we hypothesized that relationship status would explain a unique variance in sex differences in jealousy beyond biological sex and 2D:4D. As expected, a logistic regression analysis revealed that relationship status predicted F-C jealousy in males and females, indicating that both sexes in committed relationships chose their partners’ sexual infidelity as more upsetting or distressing than those not in one. Hierarchical multiple regressions also showed that relationship status predicted both sexual and emotional jealousy even when controlling for the effects of sex and 2D:4D. This result indicated that males and females in committed relationships were more upset or distressed by their partners’ sexual and emotional infidelity than those not in one. A series of these findings were consistent with previous studies (e.g., Becker et al., 2004; Guadagno and Sagarin, 2010; Kato, 2014a,b, 2021) and also supported our hypothesis concerning forced-choice and continuous measurements. These findings implied that sex differences in jealousy might emerge from relationship status.

Furthermore, our hypothesis that the effect of relationship status on jealousy would be observed, especially in sexual jealousy, was supported as the effect size of relationship status in sexual (large) was greater than that in emotional jealousy (medium). A discussion of these findings follows later in this paper.

### 2D:4D

The right 2D:4D in males was lower than that in females in the present study. This result was consistent with previous studies (see Hönekopp and Watson, 2010, for a review). However, the effect size in our study (*d* = 0.17) was minimal compared to a meta-analysis (*d* = 0.35; Hönekopp and Watson, 2010) on 2D:4D using direct measurement.

Surprisingly, the 2D:4D of participants in committed relationships was higher than those not in one. To our knowledge, no study has examined the association between 2D:4D and relationship status. This finding may be interpretable from an evolutionary psychological perspective. However, the effect size of the association between 2D:4D and relationship status was small in the present study. Thus, this association may be simply due to chance. Smoliga et al. (2021) study, published in the British Medical Journal, found a significant correlation between right 2D:4D in men and good luck (i.e., poker hand rank from randomly selected playing cards as a surrogate). Their finding was not meant to provide confirmatory evidence for the association between 2D:4D and good luck. Instead, it confirmed that the association was simply due to chance. This issue is also addressed in the limitations section.

The present study observed a slight sex difference in 2D:4D and an unexpected result regarding the association between 2D:4D and relationship status. However, it also may provide valuable data for 2D:4D research. Direct measurement used in the present study is more costly in terms of participant time than indirect measurement. Notably, many studies have used indirect measurement (Ribeiro et al., 2016). Nonetheless, our sample was relatively large comparing to those in most 2D:4D studies using direct measurement. To our knowledge, it was the largest among the 2D:4D studies at least in Japanese, including indirect measurement. Given our data’s importance, statistics on sex differences in 2D:4D are provided in the Supplementary material.

### EJM hypothesis and relationship status

The EJM hypothesis was supported only by a simple χ^2^-test on a forced-choice measure. The effect size was negligible. According to the gender similarities hypothesis (Hyde, 2014), small effect sizes like this study’s may indicate a similarity in jealousy instead of a sex difference. Additionally, a logistic regression analysis revealed that the sex difference in jealousy was not observed in participants in committed relationships. This finding was consistent with previous studies (e.g., Becker et al., 2004; Guadagno and Sagarin, 2010; Kato, 2014a,b, 2021).

Furthermore, in a continuous measurement paradigm, no sex differences were found in sexual and emotional jealousy. Our findings on a continuous measure were inconsistent with the predictions of the EJM hypothesis. However, the meta-analyses on sex differences in jealousy using a continuous measurement have replicated the different results from the predictions of the EJM hypothesis (see Kato, 2022b, for a review). Therefore, our findings on a continuous measurement are likely valid.

These findings on the EJM and our hypothesis suggest that sex differences in jealousy may emerge from sociocultural factors, such as relationship status, rather than innate mechanism. The gender similarities hypothesis (Hyde, 2014) proposes that males and females are similar in most psychological variable. Sex differences in jealousy may be one of these. Our findings, which cast doubt on the EJM hypothesis, may help clarify sex differences (or similarities) in jealousy. Moreover, they advance research on sociocultural factors regarding sex differences in jealousy. Research on sex differences in jealousy has been dominated by evolutionary psychological findings based on the EJM hypothesis. Furthermore, the EJM is a core hypothesis of evolutionary psychology. Findings based on EJM are crucial evidence for other evolutionary psychology perspectives. Currently, evolutionary psychological perspectives continue to strongly influence research regarding sex differences in jealousy. Research on sex differences (or similarities) in jealousy using sociocultural perspectives has been conducted primarily by evolutionary psychology skeptics.

It should be noted that sex differences in jealousy might be explained by other sociocultural factors not measured in this study, as well as to relationship status. Furthermore, another sociocultural factor may explain sex differences in jealousy better than relationship status. For example, the sexual imagination hypothesis predicts that sex differences in jealousy are not observed when individuals can imagine explicitly and vividly their partners’ infidelity (especially sexual imagination). On the other hand, relationship status is one factor enhancing their sexual and emotional imaginations (Kato, 2014a, 2022b). It is not a direct cause of sex differences in jealousy. Rather, it facilitates the emergence of sex differences in jealousy through being mediated by imagining a partner’s infidelity. Further studies examining the association between relationship status and sexual (or emotional) imagination might elucidate the role of the sexual imagination hypothesis in sex differences (or similarities) in jealousy. In this instance, measuring sociocultural factors other than relationship status that may affect the imagination of a partner’s sexual and emotional infidelity is required; for example, experiencing a partner’s infidelity.

### An evolutionary psychological perspective on 2D:4D

An evolutionary psychological perspective predicts that lower 2D:4D is associated with stronger sexual jealousy in males and females. However, our findings indicate that 2D:4D is not associated with sexual (or emotional) jealousy in both sexes. They were inconsistent with two previous studies (i.e., Fussell et al., 2011; Bendixen et al., 2015). This inconsistency may result from differences between the present investigation and two other studies. First, we measured finger lengths directly, while the previous studies measured them indirectly. Second, the effect size (*d* = 0.17) of the sex difference in right 2D:4D in our study was comparatively smaller than that (*d*s = 0.28 and 0.57) in the previous studies. However, replicating the findings of the two previous studies regarding the association between 2D:4D and jealousy will be difficult. The number (*N* = 1,572) of participants in our study is greater than that (*N*s = 480 and 280) in previous studies. Furthermore, the direct measurement used in the present study is suitable for assessing 2D:4D. It is not appropriate for the indirect measurement used in the previous studies (Ribeiro et al., 2016). This concern is discussed below in the limitations section.

## Limitations

This study has some other limitations. First, our hypotheses were formulated based on the sexual imagination hypothesis; however, the present study did not examine the association of relationship status with the sexual imagination hypothesis. Some studies (e.g., Becker et al., 2004; Kato, 2014a, 2021) found that individuals in committed relationships could imagine their partners’ infidelity more vividly and easily, especially sexual infidelity, compared to individuals not in one. A more detailed examination of the association between relationship status and sexual imagination might help clarify how sex differences in jealousy depend on relationship status.

Second, our findings cast doubt on the EJM hypothesis. However, they do not completely debunk it. The present study was not designed to discredit the EJM hypothesis. However, such studies will eventually determine its validity.

Third, our study failed to detect an association between 2D:4D and jealousy. However, its results were inconsistent with two previous studies. However, in recent years, skepticism concerning the relationship between 2D:4D and psychological characteristics, such as personality, cognitive abilities, and behavioral traits, has been repeatedly raised (see Leslie, 2019). Additionally, even if there is any association between 2D:4D and jealousy, other interpretations from an evolutionary psychological perspective may exist. Therefore, the relationship between 2D:4D and jealousy must be interpreted cautiously. Moreover, further studies measuring, other biological mechanisms related to the EJM instead of 2D:4D may effectively test this hypothesis.

Fourth, though small, the effect size indicates an association between 2D:4D and relationship status. The present study did not test this association based on any hypothesis. No previous study has examined this association. The association might be due to chance, according to Smoliga et al. (2021). However, the present study’s findings may be interpretable from an evolutionary psychological perspective, although we could not conceive of its interpretation. Further studies need to examine the association between 2D:4D and relationship status based on a reasonable hypothesis.

Finally, this study measured only relationship status as a sociocultural factor for sex differences in jealousy. However, multiple studies have supported sociocultural perspectives that differ in their theoretical backgrounds, such as those involving sex roles (Hupka and Bank, 1996), social cognitive (White and Mullen, 1989), and biosocial theories (Wood and Eagly, 2002). Further research adding these factors would contribute to our understanding of the causes of sex differences in jealousy.

## Conclusion

Although our study has a few limitations, it confirms that individuals in committed relationships were more upset or distressed by their partner’s infidelity, especially sexual infidelity, compared to those not in one. Moreover, relationship status explained a unique discrepancy in jealousy beyond biological sex and 2D:4D. These findings imply that sex differences in jealousy are influenced by sociocultural factors, such as relationship status, and responses to partners’ infidelity are similar. Our findings contribute to advancing research on sex differences in jealousy from a sociocultural perspective. Furthermore, our findings supported the sexual imagination hypothesis indirectly and cast doubt on evolutionary psychological perspectives.

## Data availability statement

The raw data supporting the conclusions of this article will be made available by the authors, without undue reservation.

## Ethics statement

The studies involving human participants were reviewed and approved by the Graduate School of Sociology, Toyo University. The patients/participants provided their written informed consent to participate in this study.

## Author contributions

TK: conceptualization, methodology, validation, formal analysis, investigation, visualization, supervision, project administration, and writing—original draft. NO: data curation, investigation, and writing—review and editing. All authors contributed to the article and approved the submitted version.

## Conflict of interest

The authors declare that the research was conducted in the absence of any commercial or financial relationships that could be construed as a potential conflict of interest.

## Publisher’s note

All claims expressed in this article are solely those of the authors and do not necessarily represent those of their affiliated organizations, or those of the publisher, the editors and the reviewers. Any product that may be evaluated in this article, or claim that may be made by its manufacturer, is not guaranteed or endorsed by the publisher.

## Supplementary material

The Supplementary material for this article can be found online at: https://www.frontiersin.org/articles/10.3389/fpsyg.2023.1158751/full#supplementary-material
