# Supplementary material for: Relationship status and gender-related differences in response to infidelity
Source: Front Psychol. 2023 May 24;14:1158751. doi: 10.3389/fpsyg.2023.1158751 (PMC10244511; doi:10.3389/fpsyg.2023.1158751)
Supplement: Supplementary file 1 [file Table_1.docx]

Supplementary Material

The influence of relationship experience on gender-related differences in response to a partner's infidelity

Tsukasa Kato*, Nobutoshi Okubo

*** Correspondence:** Corresponding Author: mtsukasa@hotmail.com

**Means and standard deviations of 2D:4D by biological sex**

|  |  |  |  |  |  |  |  |
| --- | --- | --- | --- | --- | --- | --- | --- |
| Variable | Mean | SD | Mean | SD | *t* value | *p* value | Effect size (*d*) |
|  |  |  |  |  |  |  |  |
|  | Men (*n* = 660) | | Women (*n* = 912) | |  |  |  |
|  |  | |  | |  |  |  |
| Right 2D:4D | 0.972 | 0.050 | 0.980 | 0.044 | 3.49 | <0.001 | 0.17 |
| Left 2D:4D | 0.971 | 0.044 | 0.976 | 0.046 | 2.07 | 0.039 | 0.11 |
| Dominant hand 2D:4D | 0.972 | 0.049 | 0.980 | 0.045 | 3.28 | <0.001 | 0.17 |
|  |  |  |  |  |  |  |  |
